# Supplementary figures and images for: Identification of a novel intermittent hypoxia-related prognostic lncRNA signature and the ceRNA of lncRNA GSEC/miR-873-3p/EGLN3 regulatory axis in lung adenocarcinoma
Source: PeerJ. 2023 Oct 10;11:e16242. doi: 10.7717/peerj.16242 (PMC10573295; doi:10.7717/peerj.16242)

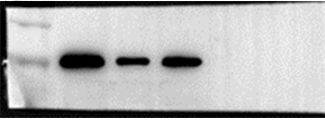

Supplement: Supplemental Information 2 [file peerj-11-16242-s002.zip › Raw data/A549-EGLN3-12.png]

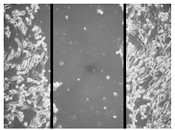

Supplement: Supplemental Information 2 [file peerj-11-16242-s002.zip › Raw data/A549-si-ctrl+ctrl-inhibitor-0h.png]

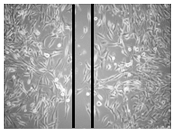

Supplement: Supplemental Information 2 [file peerj-11-16242-s002.zip › Raw data/A549-si-ctrl+ctrl-inhibitor-24h.png]

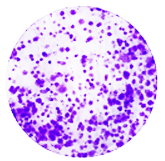

Supplement: Supplemental Information 2 [file peerj-11-16242-s002.zip › Raw data/A549-si-ctrl+ctrl-inhibitor.png]

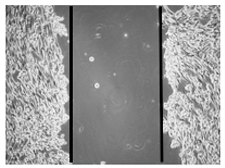

Supplement: Supplemental Information 2 [file peerj-11-16242-s002.zip › Raw data/A549-si-ctrl-0h.png]

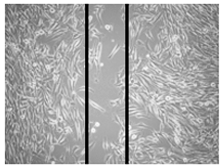

Supplement: Supplemental Information 2 [file peerj-11-16242-s002.zip › Raw data/A549-si-ctrl-24h.png]

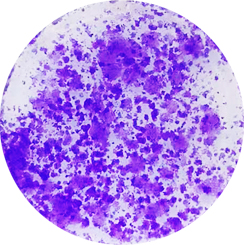

Supplement: Supplemental Information 2 [file peerj-11-16242-s002.zip › Raw data/A549-si-ctrl.png]

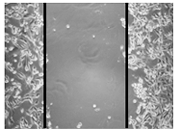

Supplement: Supplemental Information 2 [file peerj-11-16242-s002.zip › Raw data/A549-si-GSEC+ctrl-inhibitor-0h.png]

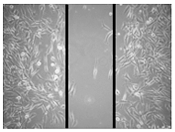

Supplement: Supplemental Information 2 [file peerj-11-16242-s002.zip › Raw data/A549-si-GSEC+ctrl-inhibitor-24h.png]

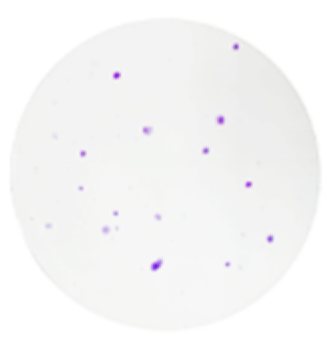

Supplement: Supplemental Information 2 [file peerj-11-16242-s002.zip › Raw data/A549-si-GSEC+ctrl-inhibitor.png]

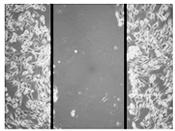

Supplement: Supplemental Information 2 [file peerj-11-16242-s002.zip › Raw data/A549-si-GSEC+miR-873-3P-inhibitor-0h.png]

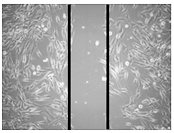

Supplement: Supplemental Information 2 [file peerj-11-16242-s002.zip › Raw data/A549-si-GSEC+miR-873-3P-inhibitor-24h.png]

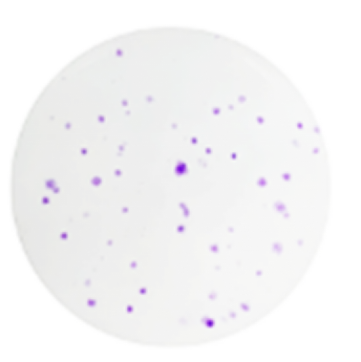

Supplement: Supplemental Information 2 [file peerj-11-16242-s002.zip › Raw data/A549-si-GSEC+miR-873-3P-inhibitor.png]

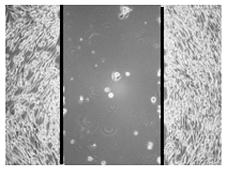

Supplement: Supplemental Information 2 [file peerj-11-16242-s002.zip › Raw data/A549-si-GSEC-0h.png]

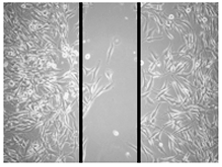

Supplement: Supplemental Information 2 [file peerj-11-16242-s002.zip › Raw data/A549-si-GSEC-24h.png]

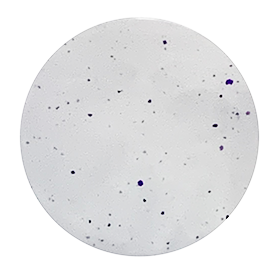

Supplement: Supplemental Information 2 [file peerj-11-16242-s002.zip › Raw data/A549-si-GSEC.png]

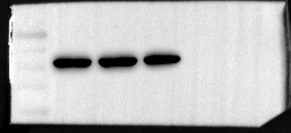

Supplement: Supplemental Information 2 [file peerj-11-16242-s002.zip › Raw data/A549-β-actin-12.png]

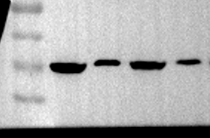

Supplement: Supplemental Information 2 [file peerj-11-16242-s002.zip › Raw data/EGLN3-12.png]

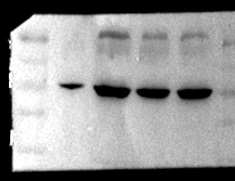

Supplement: Supplemental Information 2 [file peerj-11-16242-s002.zip › Raw data/EGLN3-2.png]

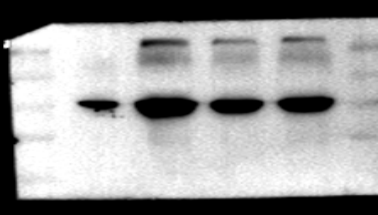

Supplement: Supplemental Information 2 [file peerj-11-16242-s002.zip › Raw data/EGLN3-3.png]

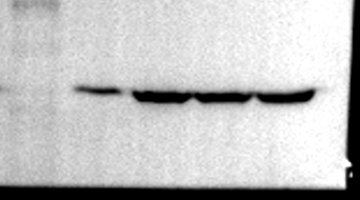

Supplement: Supplemental Information 2 [file peerj-11-16242-s002.zip › Raw data/EGLN3.png]

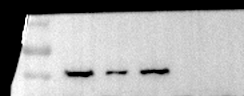

Supplement: Supplemental Information 2 [file peerj-11-16242-s002.zip › Raw data/PC9-EGLN3-12.png]

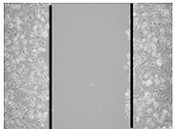

Supplement: Supplemental Information 2 [file peerj-11-16242-s002.zip › Raw data/PC9-si-ctrl+ctrl-inhibitor-0h.png]

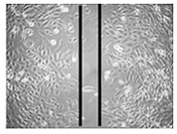

Supplement: Supplemental Information 2 [file peerj-11-16242-s002.zip › Raw data/PC9-si-ctrl+ctrl-inhibitor-24h.png]

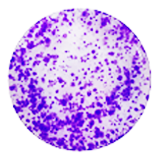

Supplement: Supplemental Information 2 [file peerj-11-16242-s002.zip › Raw data/PC9-si-ctrl+ctrl-inhibitor.png]

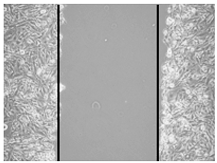

Supplement: Supplemental Information 2 [file peerj-11-16242-s002.zip › Raw data/PC9-si-ctrl-0h.png]

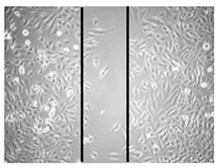

Supplement: Supplemental Information 2 [file peerj-11-16242-s002.zip › Raw data/PC9-si-ctrl-24h.png]

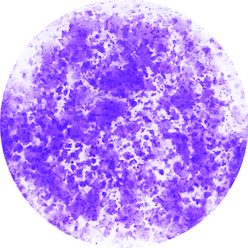

Supplement: Supplemental Information 2 [file peerj-11-16242-s002.zip › Raw data/PC9-si-ctrl.png]

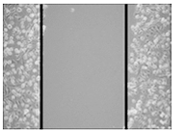

Supplement: Supplemental Information 2 [file peerj-11-16242-s002.zip › Raw data/PC9-si-GSEC+ctrl-inhibitor-0h.png]

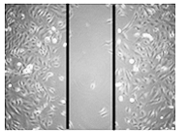

Supplement: Supplemental Information 2 [file peerj-11-16242-s002.zip › Raw data/PC9-si-GSEC+ctrl-inhibitor-24h.png]

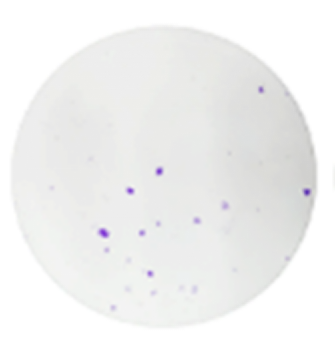

Supplement: Supplemental Information 2 [file peerj-11-16242-s002.zip › Raw data/PC9-si-GSEC+ctrl-inhibitor.png]

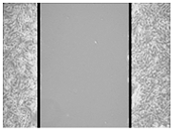

Supplement: Supplemental Information 2 [file peerj-11-16242-s002.zip › Raw data/PC9-si-GSEC+miR-873-3P-inhibitor-0h.png]

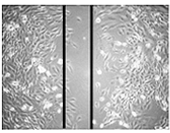

Supplement: Supplemental Information 2 [file peerj-11-16242-s002.zip › Raw data/PC9-si-GSEC+miR-873-3P-inhibitor-24h.png]

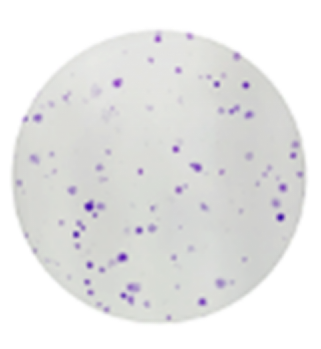

Supplement: Supplemental Information 2 [file peerj-11-16242-s002.zip › Raw data/PC9-si-GSEC+miR-873-3P-inhibitor.png]

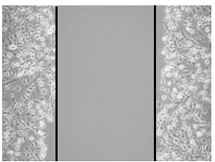

Supplement: Supplemental Information 2 [file peerj-11-16242-s002.zip › Raw data/PC9-si-GSEC-0h.png]

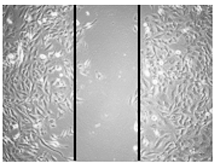

Supplement: Supplemental Information 2 [file peerj-11-16242-s002.zip › Raw data/PC9-si-GSEC-24h.png]

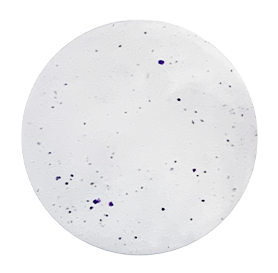

Supplement: Supplemental Information 2 [file peerj-11-16242-s002.zip › Raw data/PC9-si-GSEC.png]

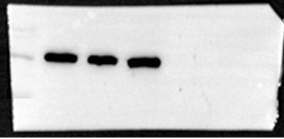

Supplement: Supplemental Information 2 [file peerj-11-16242-s002.zip › Raw data/PC9-β-actin-12.png]

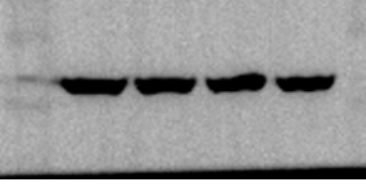

Supplement: Supplemental Information 2 [file peerj-11-16242-s002.zip › Raw data/β-actin-12-1.png]

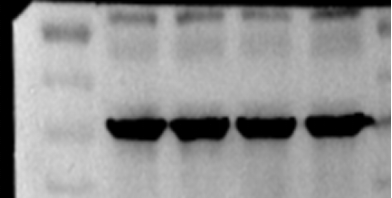

Supplement: Supplemental Information 2 [file peerj-11-16242-s002.zip › Raw data/β-actin-2.png]

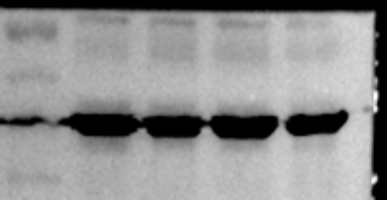

Supplement: Supplemental Information 2 [file peerj-11-16242-s002.zip › Raw data/β-actin-3.png]

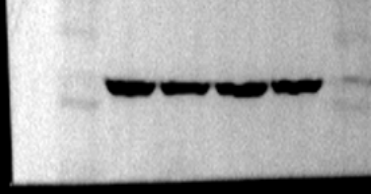

Supplement: Supplemental Information 2 [file peerj-11-16242-s002.zip › Raw data/β-actin.png]
